# Genetically Encoded Trensor Circuits Report HeLa Cell Treatment with Polyplexed Plasmid DNA and Small-Molecule Transfection Modulators

**DOI:** 10.1021/acssynbio.4c00148

**Published:** 2024-09-06

**Authors:** Chileab Redwood-Sawyerr, Geoffrey Howe, Andalucia Evans Theodore, Darren N. Nesbeth

**Affiliations:** Department of Biochemical Engineering, University College London, Bernard Katz Building, London WC1E 6BT, U.K.

**Keywords:** transient transfection, sensor, HeLa, promoter, small molecule, PEI

## Abstract

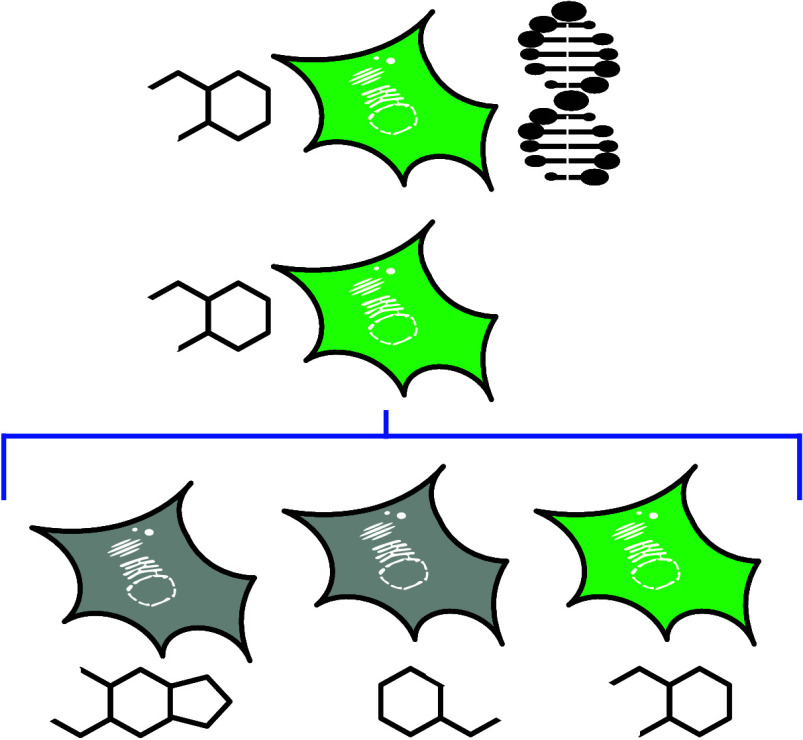

HeLa cell transfection with plasmid DNA (pDNA) is widely used to materialize biologicals and as a preclinical test of nucleic acid-based vaccine efficacy. We sought to genetically encode mammalian transfection sensor (Trensor) circuits and test their utility in HeLa cells for detecting molecules and methods for their propensity to influence transfection. We intended these Trensor circuits to be triggered if their host cell was treated with polyplexed pDNA or certain small-molecule modulators of transfection. We prioritized three promoters, implicated by others in feedback responses as cells import and process foreign material and stably integrated each into the genomes of three different cell lines, each upstream of a green fluorescent protein (GFP) open reading frame within a transgene. All three Trensor circuits showed an increase in their GFP expression when their host HeLa cells were incubated with pDNA and the degraded polyamidoamine dendrimer reagent, SuperFect. We next experimentally demonstrated the modulation of PEI-mediated HeLa cell transient transfection by four different small molecules, with Trichostatin A (TSA) showing the greatest propensity to boost transgene expression. The Trensor circuit based on the *TRA2B* promoter (Trensor-T) was triggered by incubation with TSA alone and not the other three small molecules. These data suggest that mammalian reporter circuits could enable low-cost, high-throughput screening to identify novel transfection methods and reagents without the need to perform actual transfections requiring costly plasmids or expensive fluorescent labels.

## Introduction

HeLa cells have an unmatched track record as a basic research tool^1^ and have also been explored as potential industrial production hosts for the manufacture of biologicals via transient transfection by companies including Genethon S.A.^2^ and Rentschler Biotechnologie GmbH.^3,4^ HeLa cells are also increasingly used as a preclinical testbed^5^ for characterizing transient transfection performance in the context of nucleic acid-based vaccines, by companies including Sanofi S.A. (2022 patent US2022/0142923A1) and ModernaTX, Inc. (2019 patent US10449244B2).

Commercially available methods to improve HeLa cell transfection are regularly being launched, such as the TransIT-HeLaMONSTER Transfection Kit (Mirus Bio LLC), and established reagents, such as SuperFect (Qiagen Gmbh, Germany). Development of novel, and therefore potentially proprietary, methods for improving transient transfection of HeLa, and other mammalian cell lines, can be both time-consuming and costly, due to the potentially large amounts of nucleic acid and transfection reagent needed for screening experiments.^6,7^ Here we sought to genetically encode transfection sensor (Trensor) circuits and test their utility in HeLa cells for detecting molecules and methods for their propensity to influence transfection. We anticipate that, in the future, such systems could enable low-cost and rapid screening to identify improved transfection methods and reagents.

Transfection of mammalian cells^8,9^ begins with obtaining a sufficient quantity of plasmid DNA (Figure 1.1). The pure plasmid DNA (pDNA) is typically incubated with a polyplexing compound, which helps the delivery process. The polyplexed plasmid DNA is then taken up by cells through a process that tends to involve clathrin-mediated endocytosis,^10^ where it is engulfed within endocytic vesicles. Subsequently, the plasmid is released from these vesicles and resides in the cytosol. From the cytosol, the plasmid relocates to the nucleus, where gene expression occurs, which may also be accompanied by the integration of the foreign genetic material into the host cell’s genome.

**Figure 1:**
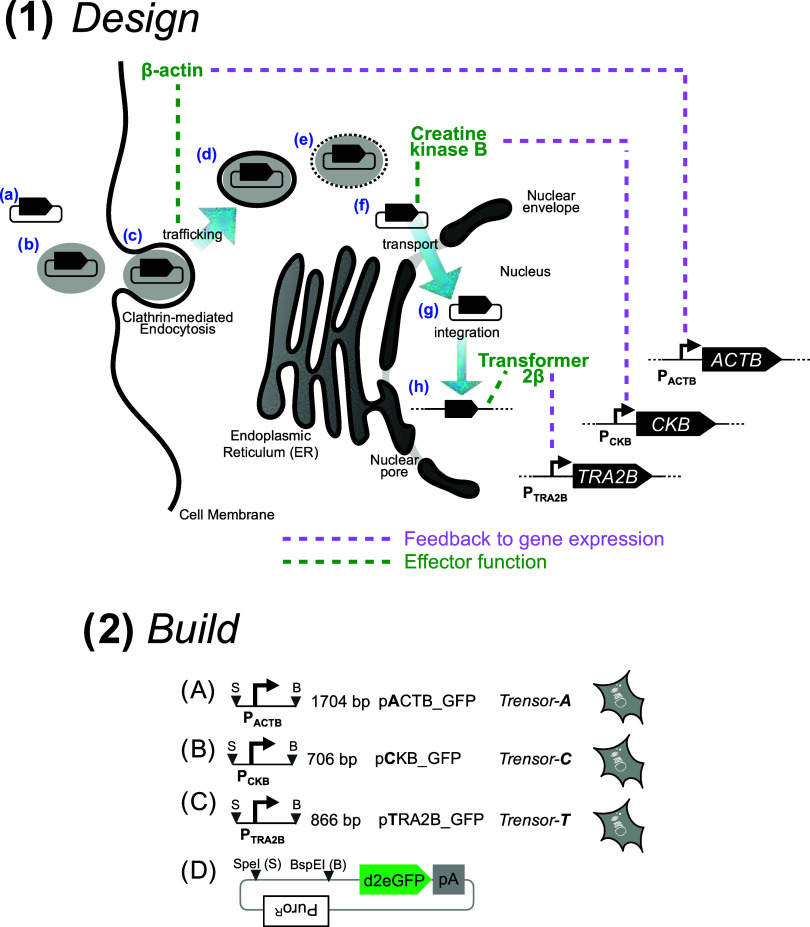
Harnessing transcriptional impacts of plasmid DNA transfection. We used observations and hypotheses to inform the design (panel 1) of a set of Trensor circuits, which we then proceeded to build (panel 2) via service-provider DNA synthesis and assembly. (Panel 1) Transfection of mammalian cells (a) begins with materializing sufficient mass of plasmid DNA, then (b) typically incubating the pure plasmid DNA with a polyplexing compound, represented here by the gray oval. Polyplexed plasmid DNA will then typically (c) be taken up by cells via clathrin-mediated endocytosis, followed by (d) pinching off into endocytic vesicles from which the plasmid will (e) ultimately be liberated to (f) reside in the cytosol. From the cytosol, the plasmid will (g) relocate to the nucleus whereupon (h) genomic integration will tend to occur at low frequency. Three proteins (green text) may influence (green dashed lines) these steps. β-Actin contributes to the cellular machinery that drives vesicular trafficking. Creatine kinase B is a cytoplasmic enzyme involved in modulating the availability of energy for processes likely to include transport of plasmid DNA to the nucleus. Transformer 2β is a nuclear protein that influences mRNA maturation for native genes, so it may mis-process mRNA arising from transgenes which are typically devoid of introns. In this report, we hypothesize that causal links (pink dashed lines), of as yet unknown mechanism, exist whereby the abundance and/or activity of each of these proteins feeds back to influence the activity of the promoters within their encoding genes. (Panel 2). Three plasmids were assembled from fragments encoding the putative promoters from the human genes that encode the three panel 1 proteins: (A) ACTB, (B) CKB, and (C) TRA2B. Each promoter-encoding fragment featured flanking SpeI (downward triangle labeled “S”) and BspEI restriction sites (downward triangle labeled “B”) for directional insertion into cognate sites present in (D) the plasmid, pGTIP. pGTIP encodes SpeI and BspEI sites, upstream of an open reading frame (ORF) encoding a short-lived green fluorescence protein, d2eGFP.^11^ Stable transfectant HeLa cell lines were named according to the Trensor circuit they harbored.

## Experimental Challenges in Characterizing Transient Transfection

Assessing the effectiveness of a given method, or set of reagents, for transfecting mammalian cells can be costly and challenging. Insights can be gained from imaging the internalization and intracellular localization of pDNA during the transfection process (Figure 1(1)). Fluorescence microscopy is one approach to achieving this visualization. Labeling with fluorescent dyes tends to be necessary for this, such as Oregon Green 488 to label the polyplex material used to deliver the plasmid DNA,^12,13^ or TOTO-3, to directly label the plasmid DNA.^14,15^ Phototoxic effects on cells and photobleaching during fluorescence microscopy can both confound the image data arising from these approaches.^16,17^ The use of reporter genes encoded by the transfected plasmid is another approach, with the limitation that transgene expression can be epigenetically impacted post-transfection, via processes such as methylation.^18,19^

### Biological Impacts of pDNA Transfection on Mammalian Cells

Transcription of the mammalian genes, ACTB, CKB, and TRA2B, is significantly perturbed in cells undergoing transient transfection^20^ using branched polyethylenimine (B-PEI), with an average MW of 22 kDa. Given this observation, we chose to investigate these promoters in preference to (i) a random selection of promoters and (ii) promoters shown to be unaffected by B-PEI transient transfection. We anticipated that any effect on the activity of these promoters, whether upregulation,^20^ or downregulation, would have utility within an engineered biosensor. Upregulation could be coupled directly to a reporter, whereas downregulation could be incorporated into a circuit where the expression of a repressor decreased, with a concomitant increase in the expression of a derepressed reporter.

ACTB encodes β-actin, one of the six actin proteins ubiquitous in mammalian cells and involved in vesicular trafficking processes. The CKB gene encodes creatine kinase B, an enzyme that contributes to the modulation of energy availability for a range of cellular processes, including intracellular trafficking.^21,22^ Energy dysregulation has been shown to arise when cells undergo transfection via PEI.^23^ TRA2B encodes Transformer 2β, a nucleus-resident protein involved in general mRNA splicing. Changes in the expression of splicing factors, such as Transformer 2β, can impact global translation levels in mammalian cells. This is particularly the case in the unfolded protein response, which results when cells exceed their capacity to properly fold the proteins they are making.^24^

Figure 1(1) illustrates our proposal that β-actin and creatine kinase B influence cytosolic transport events that are perturbed by transfection, while Transformer 2β influences the expression of the delivered transgenes via its role in mRNA maturation. There is to date no reported mechanistic data regarding the link between transfection and induction of the three genes encoding these proteins.^20^ For this study, there is a 2-fold working hypothesis. First, that these genes are autoregulated by the abundance of their gene product.^25,26^ Second, that changes in the activity of these three proteins resulting from incubation with transfection-influencing agents lead to increases or decreases in their turnover and therefore abundance. Further research will be needed to test this 2-fold hypothesis; however, we suggest that the assumption that these genes are autoregulated is reasonable given evidence from genome-wide studies that gene autoregulation is common in mammalian cells.^27,28^

### Establishing Genetically Encoded Reporters in Mammalian Cells

Genetically encoded reporters represent a unique and promising area of synthetic biology, leveraging the complex machinery of living cells for monitoring natural environments; contaminants, toxins, and intended targets, and industrial environments and processes. The reporter function can be naturally occurring or artificially introduced through synthetic biology techniques for gene and gene network design. Detection can harness the complex biological responses of the cells, including changes in metabolic activity, conformation of a given protein or nucleic acid molecule, protein synthesis, or gene expression, each of which can be used as an indirect measure of the presence and sometimes the concentration of a specific analyte.

Key components of a genetically encoded reporter include the sensing element, conversion of the biological response into a detectable signal, and a reporter system that interprets and presents the data. Reporters are typically based on a change in fluorescence, color, light production, or electrical resistance, depending on the specific design of the biosensor.

The large majority of genetically encoded reporters developed to date have been microbial.^29^ Mammalian genetically encoded reporters have been engineered at the level of transcriptional control, often via a promoter whose activity level is robustly influenced by the presence or absence of a given analyte or condition. Typically, the promoter is deployed in the transgene to control the expression of a reporter protein such as green fluorescent protein (GFP). Mammalian genetically encoded reporters have been engineered using promoters in this way to signal when a cell is experiencing mechanical stress,^30^ cellular stress due to the presence of unfolded proteins^31,32^ in the endoplasmic reticulum (ER), or stress due to hypoxia.^33^ To date, no mammalian genetically encoded reporters, that we are aware of, have been engineered to detect the occurrence of transient transfection or to detect chemicals known to modulate transient transfection.

### Small-Molecule Modulation of Transient Transfection

Small molecules can be used to modulate mammalian cell transient transfection. Examples include the ability of a range of small-molecule inhibitors of apoptosis to enhance gene transfer when treating NIH 3T3 murine fibroblasts with branched polyethylenimine (B-PEI), and pDNA.^34^ Below we discuss four small molecules with respect to their proven or potential ability to modulate transient transfection.

Hydrocortisone (HCS) has been reported^35^ to improve transient transfection of a range of immortalized, adherent murine cell lines^36^ and an immortal adherent human keratinocyte cell line.^37^ Trichostatin A (TSA) improved^38^ the performance of transient transfection of mouse embryonic stem cells performed using both FuGENE and Lipofectamine 2000 as transfection agents. TSA has also been reported to inhibit the ability of the protein, ZNF511-PRAP1, to bind plasmid DNA and inhibit transgene expression in cells undergoing transient transfection.^39^

While HCS and TSA have both been reported to improve transient transfection performance, for the purposes of this study, we also wished to test small molecules that either (i) also enhanced transient transfection or (ii) inhibited transient transfection. Our criteria for such candidate small molecules were that they reportedly interacted with similar features of mammalian cell biology as HCS and TSA (i.e., epigenetic control, cytoskeletal components, or trafficking processes).

4-Hydroxytamoxifen (4-HT) binds to estrogen receptors, an ancient class of protein present in the cells of a wide range of multicellular animals. Estrogen receptor-based pathways can govern a wide range of cellular processes,^40^ including the types of ion channel regulation that impact intracellular trafficking events. 4-HT binding to estrogen receptors has been shown to activate cell survival and growth pathways,^41^ which may also help cells withstand the stress of transient transfection. S-[(6S)-7-(1-adamantylamino)-6-[(2-methylpropan-2-yl)oxycarbonylamino]-7-oxoheptyl] 2-methylpropanethioate (TCS) inhibits the expression^42^ of the gene encoding histone deacetylase 6 (HDAC6), a protein involved in microtubule-dependent cytoskeleton rearrangement.^43^Figure 3(1) depicts our hypothesis that TCS, HCS, TSA, and 4-HT impact cellular processes in a manner that affects the performance of transfection procedures.

### Genetically Encoding Transfection Sensor “Trensor” Circuits in Mammalian Cells

An ideal genetically encoded encoding transfection sensor “Trensor” circuit would harness cellular events that arise as a result of the cells being incubated with compounds known to implement or influence transient transfection. Such Trensor circuits could potentially act as a screening platform to identify previously unknown methods or compounds that affect or enhance transfection.

In this study, we sought to genetically encode three transfection sensor (Trensor) circuits and test their utility in the HeLa cell line (Figure 1(2)). We first tested whether these Trensor circuits would be triggered if their host cells were treated with pDNA and Superfect. We next tested four small molecules for their ability to enhance or inhibit transgene expression in unmodified HeLa cells after their treatment with pDNA and linear polyethylenimine of 25 kDa average molecular weight (L-PEI). We then determined the general sensitivity of each Trensor circuit to each of each of these four small molecules alone.

## Materials and Methods

All novel cell lines developed during this study are available from the corresponding authors upon reasonable request. The availability of these cell lines is subject to the signing of a material transfer agreement (MTA) to ensure compliance with ethical guidelines and to maintain the scientific integrity of the materials. MTAs with University College London will typically outline terms and conditions for the use of the material and contain confidentiality and intellectual property rights provisions. The purpose of this agreement is to protect both the provider and recipient and prevent misuse of the materials. Please direct all requests to the corresponding author, and upon receipt of a request, we will initiate the MTA process. We commit to fulfilling the requests in a timely manner, subject to approval by our institutional review board. The researchers who receive the cell lines will be requested to cite this publication in any communication or publications that result from the use of these materials. All data are available upon reasonable request. Plasmid sequence data is available at Figshare doi.org/10.6084/m9.figshare.23695302.^44^

### Trensor Circuit Plasmid Design

Several genes have been identified, whose expression in HEK293T cells responds to B-PEI-mediated transient transfection.^20^ We arbitrarily selected three of those genes, ACTB, CKB, and TRA2B, as a source of putatively transfection-sensitive promoters for use in engineering Trensor circuits. We queried the ENSEMBL^45^ and UCSC^46^ genome browsers to identify transcription factors binding sites within the first 1 kb of sequence upstream of the relevant open reading frame (ORF) start codon. In this way, we identified the ORegAnno^47^ database (“OREG”) entries for putative promoter sequences for ACTB (OREG1220749), CKB (OREG16873772), and TRA2B (OREG1173020).

Each putative promoter sequence was inserted upstream of a GFP ORF encoded within a mammalian expression plasmid (Figure 1(2)), to generate the following plasmids: pACTB_GFP, encoding the putative ACTB promoter, pCKB_GFP, encoding the putative CKB promoter, and pTRA2B_GFP, encoding the putative TRA2B promoter (GenScript, New Jersey). The resultant sequencing data for each of the three assembled, plasmid-encoded genes were made available on the Figshare public data repository.^44^

### Plasmid Propagation and Isolation

Standard molecular biology techniques were used for plasmid propagation, using *Escherichia coli* (E. coli), plasmid isolation, and plasmid characterization. The three DNA fragment insertions set out in Figure 1(2) were performed by Genscript (New Jersey). All sequence data are available upon request, via the thesis document of C.R-S., deposited at https://discovery.ucl.ac.uk and in the Figshare repository^41_25^.

### HeLa Cell Cultivation and Flow Cytometry

HeLa cells, obtained from ATCC, were grown using routine methods. 10% v/v fetal bovine serum (FBS) in high-glucose/GlutaMAX Dulbecco’s modified Eagle’s medium (DMEM) media (Life Technologies, Thermo Fisher Scientific, Waltham, MA) was used throughout. T175 flasks with vented caps (Corning Limited, Union City) were used for cell growth unless otherwise stated. Cells were typically seeded at 2 × 10^5^ cells/mL and growth vessels housed in static incubators (170AICUVD, PHCbi, Tokyo, Japan) at 37 °C, 5% CO_2_ until they reached 80% confluence before being subcultured.

For cytometric analysis, cells were rinsed with typically 10 mL of phosphate-buffered saline (PBS), treated with 2 mL of trypsin-ethylenediaminetetraacetic acid (EDTA), sourced from Merck-Millipore, Germany, at 37 °C for 5 min, after which an additional 2 mL of PBS was added to resuspend the cells. Following this, the cells were centrifuged and resuspended in 1–2 mL of PBS to eliminate any remaining trypsin. Fluorescence data were acquired using a BD Accuri C6 Plus (BD Biosciences) instrument, counting 20,000 events using the FL1-A laser, which can identify the 509 nm emission peak of GFP.

### HeLa Cell Transient Transfection

For the Superfect (Qiagen, Maryland) reagent, HeLa cells were seeded at 5 × 10^5^ cells/mL to yield a confluency of approximately 75% after overnight growth. Media was changed to serum-free Ultraculture media (Lonza, U.K.), and Superfect (Qiagen, MD) plus plasmid DNA mixture added to media dropwise, as per manufacturer’s instructions. Briefly, cells were incubated with the transfection mixture for 3 h before the mixture was removed and replaced with serum-containing media until analysis by flow cytometry.

For transient transfections using linear polyethylenimine of 25 kDa average molecular weight (L-PEI), HeLa cells were seeded as above. The required mass of plasmid DNA was mixed with the required mass of L-PEI, diluted with unsupplemented DMEM, incubated at room temperature for 10 min, and administered to cells. Small-molecule additions were used to supplement L-PEI-mediated transient transfection procedures, at the concentrations indicated in Figure 3(2), 1 h prior to transfection.

### HeLa Cell Stable Transfection

Each plasmid was used to stably transfect HeLa cells as described (Materials and Methods section) and polyclonal, puromycin-resistant populations were maintained using standard tissue culture techniques. The resulting, stable transfectant cell lines were referred to as Trensor-A, stably transfected with pACTB_GFP, Trensor-C, stably transfected with pCKB_GFP, and Trensor-T, stably transfected with pTRA2B_GFP.

Stable HeLa cell transfection was performed using L-PEI in the same manner as transient, except that 5 days post-transfection puromycin was added to growth media at 1.25 μg/mL. Cell confluency was estimated every 2 days, alongside a complete change of selective media every 2 days. Untransfected negative control cells typically died after 6 days. Day 6 post-transfection was also typically the first day that puromycin-resistant growth foci were observed for transfections where the plasmid was included. All growth foci on a given plate were trypsinized, pooled, and cultivated as a polyclonal population throughout this study. The resultant, polyclonal, puromycin-resistant cell lines were each named according to the Trensor circuit they harbored.

### Trensor-Harboring Cell Analyte Incubation

Cell lines stably transfected with Trensor circuits were propagated in 10% v/v FBS DMEM and typically seeded at 2 × 10^5^ cells/mL to achieve 70–80% confluence the next day during passaging. 24 h prior to flow-cytometric analysis, the small molecules indicated in Supporting Figures 1–3 were added to growth media at the indicated concentrations. Mode fluorescence values from the data are plotted in Supporting Figures 1–3 and individually plotted in Figure 4.

## Results

We first sought to test the hypothesis that three different genetically encoded Trensor circuits could respond to their host cells undergoing treatment with pDNA and SuperFect. Second, we tested whether certain small molecules could influence (inhibit or enhance) transgene expression resulting from the treatment of unmodified HeLa cells with pDNA and L-PEI. Finally, we tested whether genetically encoded Trensor circuits could respond to those small molecules in a manner that tracks their propensity to influence transgene expression resulting from pDNA and L-PEI treatment HeLa cells. We tested these hypotheses in order to validate the Trensor circuit concept, so it can then be further characterized and applied by the wider research community who seek to improve the efficacy and/or lower the costs of stable and transient transfection procedures.

### Promoters for β-Actin, Creatine Kinase B, and Transformer 2β Respond to Treatment with SuperFect and an Antibody-Encoding Plasmid

**Figure 2:**
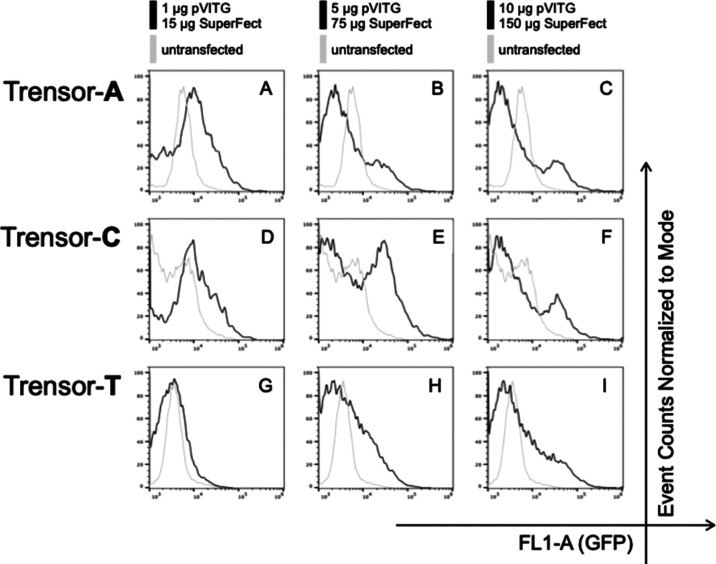
Trensor circuit response when host HeLa cells are treated with Superfect plus pDNA. HeLa cell lines harboring Trensor-A (plots A–C), Trensor-C (plots D–F), or Trensor-T circuits (plots G–I), were subjected to treated with SuperFect and the plasmid, pVITRO1-Trastuzumab-IgG4/κ (abbreviated to pVITG in the diagram for graphical brevity), as described in the Materials and Methods section. The total mass of SuperFect and pVITRO1-Trastuzumab-IgG4/κ used in each treatment is indicated above a column of data plots and was used for each data plot in that column. The level of fluorescence in untreated cells is shown in the gray fluorescence profile in each data plot, and the level in treated cells in the black fluorescence profile. Increasing total mass of SuperFect and pVITRO1-Trastuzumab-IgG4/κ are indicated above the column of plots, along with bars as a key for the gray and black data profiles in each column. *Y* and *X* axes labels for all plots are indicated in the bottom right of the figure. Data are all from experiments performed in singlicate due to the discontinuation of SuperFect as a commercial product.

SuperFect has, since the 1990s been an effective reagent for transient transfection, predominantly in research settings. The SuperFect reagent design is based on the observation that complexes formed between partially degraded cationic polyamidoamine dendrimers and DNA can be effective for plasmid transfer and subsequent transgene expression in mammalian cells.^48^ We sought to establish whether three Trensor circuits, Trensor-A, Trensor-C, and Trensor-T (Figure 2), would respond to their host cells being treated with pDNA and SuperFect. We treated the three Trensor-harboring HeLa cell lines by incubating them with SuperFect and the plasmid, pVITRO1-Trastuzumab-IgG4/κ (Addgene entry 61887), which has an extensive track record of use within mammalian cell transient transfection procedures to produce a recombinant monoclonal antibody.

Cells harboring Trensor-A (Figure 2A–C), Trensor-C (Figure 2D,E), and Trensor-T (Figure 2G,I) all responded to pDNA and SuperFect treatment by increasing their levels of green fluorescence. The data reported in Figure 2 were all captured from single treatment procedures. Unfortunately, commercial production of the SuperFect reagent ceased during this study before repeats or antibody yield experiments were possible. While other reagents based on partially degraded cationic polyamidoamine dendrimers may be available, the precise formulation used in commercial products is often proprietary, therefore confidential, information. As such, it would not be possible to perform further matched repeats using different products. Nevertheless, we suggest that there is value in sharing the data set in Figure 2 to guide future studies, beyond the scope of this current work, in building genetically encoded reporters to detect transfection mediated by partially degraded cationic polyamidoamine dendrimers.

### Fluorescence Levels Resulting from Incubation of HeLa Cells with a GFP Expression Plasmid and L-PEI Can Be Influenced by the Presence of Small-Molecule Additions

**Figure 3:**
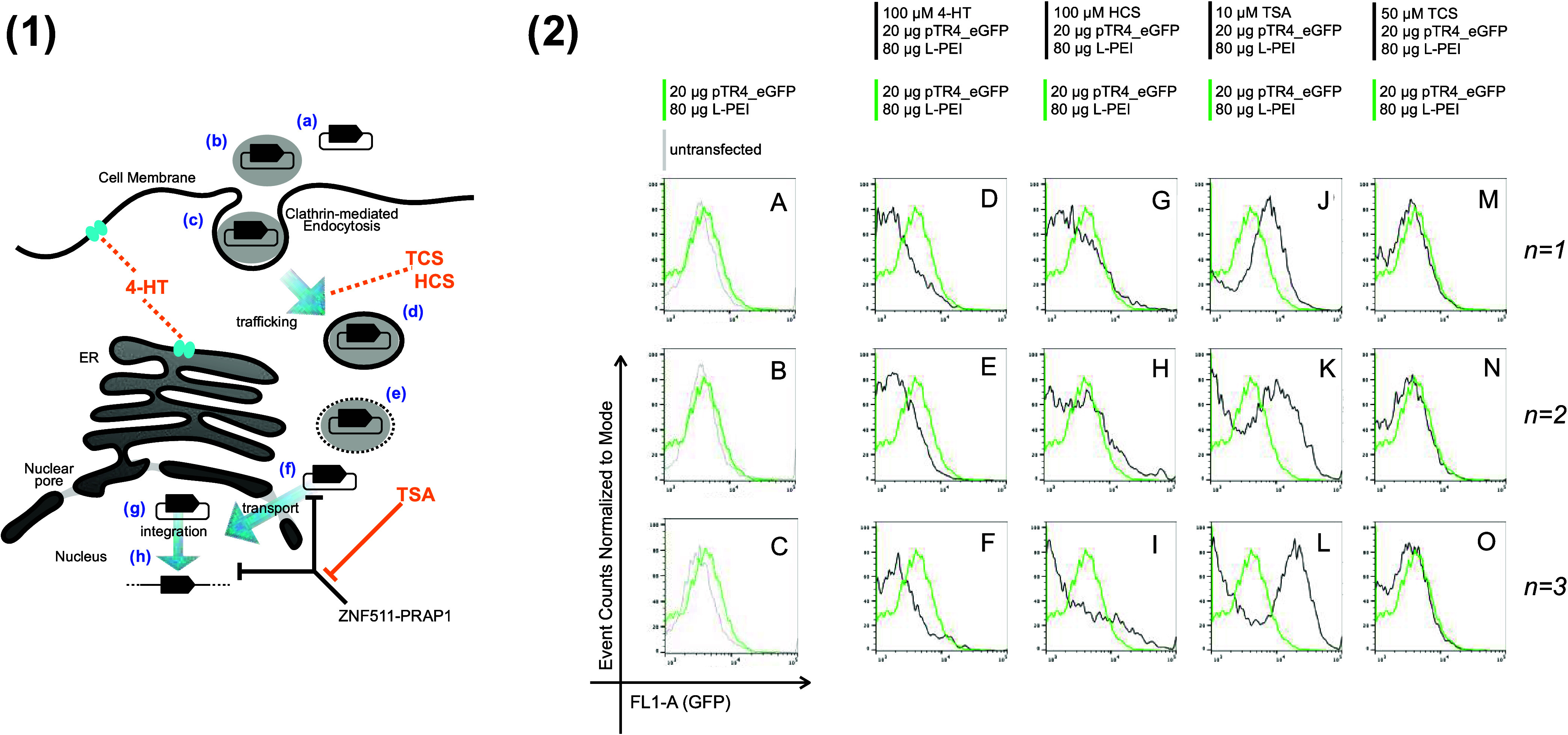
Influence of 4-HT, HCS, TCS, and TSA on transient transgene expression during incubation with plasmid DNA and L-PEI. (Panel 1) Steps in the plasmid transfection process are illustrated and annotated as in Figure 1(1) except for the following. We hypothesized that four small molecules (orange text) may influence the indicated steps either indirectly (orange dashed lines) or via mechanism involving inhibition (orange flathead arrow). 4-HT can indirectly influence ion channel (light blue graphic) activity in a manner that impacts trafficking processes. HCS and TCS each could indirectly influence intracellular trafficking levels. TSA inhibits ZNF511-PRAP1, a protein known to inhibit the expression of plasmid-encoded genes during transfection. (Panel 2) Unmodified HeLa cells were treated with four different small molecules, L-PEI and the plasmid, pTR4_eGFP, as described in the Materials and Methodssection. Green fluorescent profiles from three independent repeats of each procedure, indicated by *n* = 1, *n* = 2, and *n* = 3 on the right of each row, were plotted. The level of fluorescence of untreated cells is shown in the gray data profile in plots (A–C). For plots (D–O), the fluorescence of cells treated with small molecule, L-PEI, and pDNA is in black. For all plots, the fluorescence of cells treated with L-PEI and pDNA only is in green. *Y* and *X* axes labels for all plots are indicated in the bottom left of the figure. Treatments with 4-HT, HCS, TSA, and TCS have supplement concentrations indicated at the top of each column.

4-HT, HCS, and TCS can all indirectly modulate intracellular trafficking in mammalian cells, while TSA has been shown to directly inhibit ZNF511-PRAP1, a protein complex known to suppress the expression of genes encoded by plasmid vectors during the transfection process. Figure 3(1) depicts our hypothesis that 4-HT, HCS, and TSA influence transient transfection by affecting the trafficking events necessary for endocytosis and subsequent trafficking of endocytic vesicles. Figure 3(1) also illustrates our hypothesis that inhibition of ZNF511-PRAP1 by TSA will in turn reduce the inhibitory effects ZNF511-PRAP1 has been proven to have on transfection.

Extensive further research will be needed to fully delineate the mechanism by which these four small molecules modulate transfection performance. As a first test of these hypotheses (Figure 3(1)), we performed a procedure where unmodified HeLa cells were incubated with a GFP-encoding reporter plasmid and L-PEI (Materials and Methodssection) in the presence or absence of each of these small molecules. Subsequent plasmid-encoded reporter expression was then measured to determine if the small molecule had an inhibitory or enhancing affect.

#### TCS and 4-HT Inhibit the Performance of L-PEI and pDNA Treatment

We observed that an experimental plasmid, pTR4_eGFP, obtained from UCL Biochemical Engineering Department and encoding GFP expression, performed poorly within an L-PEI treatment with (Materials and Methodssection), with a 20 μg procedure yielding only a marginal increase in green fluorescence (Figure 3(2)A–C, green data profile) compared to untransfected HeLa cells (Figure 3(2)A–C, gray data profile).

The presence of 100 μM 4-HT during pTR4_eGFP transient transfection caused a marked reduction in fluorescence in HeLa cells (Figure 3(2)D–F, black data profile) compared with when the same transfection was performed in the absence of 4-HT (Figure 3(2)D–F, green data profile). The presence of 50 μM TCS also reduced the fluorescence signal (Figure 3(2)M–O), but to a lesser extent than 100 μM 4-HT.

#### HCS Marginally Enhances Fluorescence Arising from Treatment with L-PEI and Plasmid DNA

The presence of 100 μM HCS (Figure 3(2)G–I, black data profile) had a definite, if marginal, enhancing effect on the fluorescence arising from the pTR4_eGFP plus L-PEI treatment, compared to the HCS-free control (Figure 3(2)G–I, green data profile). Although the effect was marginal, it was consistent over *n* = 3 biological repeats.

#### TSA Enhances Fluorescence Arising from Treatment with L-PEI and Plasmid DNA

We next supplemented the L-PEI plus plasmid DNA treatment with 10 μM TSA and analyzed the fluorescence in HeLa cells. TSA provided a noticeable enhancing effect on the fluorescence output of the HeLa cells (Figure 3(2)J–L, black data profile). This enhancement contrasted with the control, unsupplemented procedure (Figure 3(2)J–L, green data profile). The presence of increased fluorescence, which is an indicator of heightened GFP expression, signifies that the TSA effectively amplifies the output of the pTR4_eGFP plus L-PEI treatment.

The consistency of the enhancing effect of TSA on fluorescence was further affirmed by performing *n* = 3 biological repeats to ensure the reproducibility and reliability of the observed results. Each replicate was independent and performed under the same experimental conditions to reduce the variables and potential bias. In each of these replicates, the enhancement effect of TSA supplementation on HeLa cell fluorescence remained evident.

### Ranking the Propensity of Four Small Molecules to Influence Treatment with L-PEI and Plasmid DNA

The data plotted in Figure 3(2) allowed us to evaluate and comparatively rank the four small-molecule compounds for their propensity to reduce or augment the fluorescent arising HeLa cell treatment with plasmid and the transfection agent, L-PEI. TSA demonstrated the most pronounced augmentative effect, followed by HCS, then TCS, with 4-HT ranked lowest. It is noteworthy that while HCS exhibited only a marginal enhancement across two out of the three experimental replicates (Figure 3(2)G–I), TCS essentially lacked any appreciable enhancement capacity, while 4-HT manifested an inhibitory influence on the fluorescence signal.

### Genetically Encoded Reporters, Trensor-A, and Trensor-C Are Not Generally Sensitive to Small-Molecule Transfection Modulators

We next sought to test whether the “Trensor” genetically encoded reporters are generally sensitive to the small-molecule transfection-influencing reagents alone, outside the context of a transient transfection experiment. Supporting Figures 1 and 2 together show that Trensor-A and Trensor-C do not markedly respond to any of the four small molecules alone and certainly not in a manner that reflects their propensity to enhance L-PEI-mediated transient transfection.

### Genetically Encoded Reporter, Trensor-T, Is Generally Sensitive to Small-Molecule Transfection Modulators

When the genetically encoded reporter, Trensor-T, was incubated with each of the four small molecules alone, reporter expression in response to TSA was clear to observe across *n* = 3 biological (Supporting Figure 3G–I). For the other three molecules, no consistent increase in Trensor-T GFP expression was observed. We concluded that in this way, Trensor-T was generally triggered only by TSA, the only small molecule that had a clear and unambiguous transfection-influencing effect.

**Figure 4:**
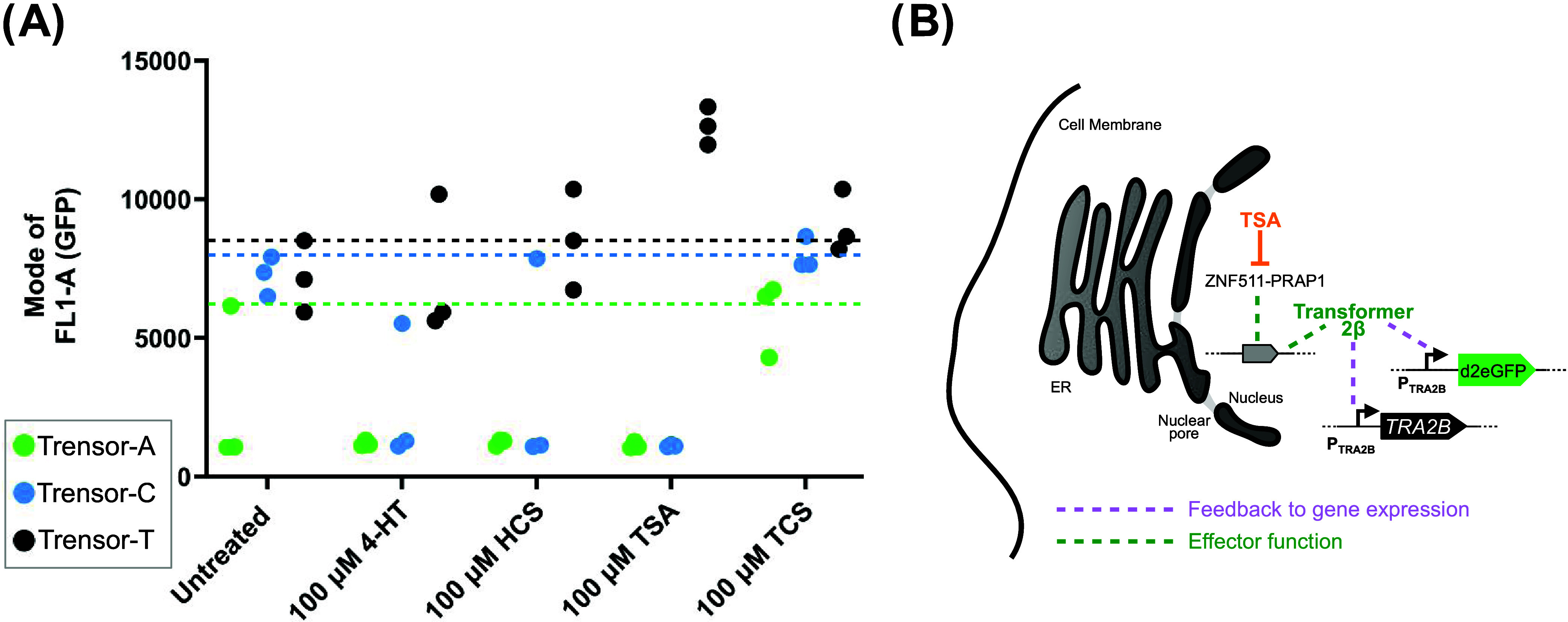
Trensor-T circuit response when host HeLa cells are treated with TSA in the absence of polyplexed pDNA. (A) Mode fluorescence values for *n* = 3 biological repeats were plotted for HeLa cells harboring Trensor-A, Trensor-C, or Trensor-T circuits and incubated with no compound (Untreated), or with 4-HT, HCS, TCS, or TSA at the indicated concentrations, in the absence of polyplexed pDNA. For untreated cells harboring Trensor-A, the highest mode fluorescence value is indicated with a green, horizontal dashed line, with equivalent blue and black dashed lines, respectively, for untreated cells harboring Trensor-C and Trensor-T circuits. (B) Graphic to illustrate our hypothesis for how the Trensor-T circuit responds to TSA. In the absence polyplexed pDNA treatment, ZNF511-PRAP1 (black text) influences only native gene expression (green dashed line to gray gene graphic). This influence is perturbed by the presence of TSA (orange text and flathead arrow). This perturbation may have a downstream effect on the activity of the nucleus-resident splicing factor, Transformer 2β, a protein that also influences (green dashed line) native gene expression. We hypothesize that causal events (pink dashed lines), of as yet unknown mechanism, link abundance and/or activity of Transformer 2β and the activity of its promoters, present in both the native TRA2B gene and the Trensor-T circuit transgene.

We next collated the data from Supporting Figures 1–3 and plotted individual mode fluorescence values for each of n = 3 biological repeats for Trensor-harboring HeLa cells either untreated or treated with each of the four small molecules (Figure 4A). Considering only mode fluorescence values allowed us to still glean insight from this particular data set, despite the fact that the polyclonal populations showed a broadening of both up- and downregulatory response that dampened differences in mean fluorescence (Supporting Figures 1–3). We defined a limit of detection (LOD) for the general response of a polyclonal population of HeLa cells harboring a given Trensor circuit as that general response in which all of at least *n* = 3 biological repeats of small-molecule treatment yielded higher mode fluorescence than the highest mode fluorescence measured over at least n = 3 biological repeat measurements of untreated cells. Only TSA treatment of HeLa cells harboring the Trensor-T circuit exceeded this LOD (Figure 4A). Figure 4B illustrates our hypothesis regarding how the Trensor-T reporter gene is induced following cell treatment with the TSA.

## Discussion

We constructed three candidate “Trensor” circuits, Trensor-A, Trensor-C, and Trensor-T, in HeLa cells (Figure 1(2)) with the intention that they could (i) respond directly to incubation with polyplexed pDNA and (ii) respond to incubation only with small molecules shown to influence transient transfection. All three candidate “Trensor” circuits were stimulated when their host cells were treated with Superfect plus pDNA (Figure 2), with the magnitude of GFP expression response tending to be influenced by the concentration of Superfect and plasmid DNA. Further study is now warranted to establish if these three Trensor circuit-harboring HeLa cell lines can be useful tools for the investigation or screening of further transfection reagents and plasmids.

All cell lines tested in this study were polyclonally derived populations, so it is unlikely that the phenotypes that have manifested have arisen due to chromosomal insertion site effects. As the Trensor-harboring cell lines are polyclonal populations, the fluorescence shifts in Figure 2 may be due to straightforward genotypic variation or may arise due to stochasticity in gene expression,^49^ a phenomenon that has been observed to manifest even against isogenic population backgrounds.^50^ In the case of genetic variation, this would suggest it may be possible to isolate clonally derived populations (“clones”) with a more marked response to stimulation (either in up- or downregulation of GFP) than the parental, polyclonal populations. If these response phenomena are stochastic in nature, then the utility of the Trensor populations would still be preserved, with the response to stimulus possibly being more robust than that which would arise from a more tightly deterministic gene regulation system.^51^

We next observed that small molecule TSA can enhance the transgene expression that results from treating unmodified HeLa cells with L-PEI and pDNA (Figure 3(2)J–L). By contrast, the small molecule HCS had only a marginal enhancement of the same treatment (Figure 3(2)G–I), despite its reported enhancement of transfection of murine cells and human keratinocyte cells.^36,37^ This suggests the pathways involved in enhancing, and sensing, transfection procedures may be cell-type-specific. 4-HT was inhibitory (Figure 3(2)D–F), which was particularly unexpected given its reported ability to bind estrogen receptors and potentially act as a hormone mimic and enhance cell viability.^41^ This may indicate that cellular health and robustness negatively correlate with transfectability. TCS was also inhibitory (Figure 3(2)M–O), to a lesser extent than 4-HT, suggesting either its presence did not affect the cytoskeleton, or did not affect it to an extent that impacted transfection performance.

We had observed that the four small molecules tested in Figure 3(2) were able to modulate transfection of regular HeLa cells, enhancing, inhibiting, or having a minimal effect. As such, we incubated HeLa cells harboring Trensor-A, Trensor-C, or Trensor-T circuits with each of the small molecules alone, outside of the context of a transfection procedure. When incubated with the small molecules only, HeLa cells harboring Trensor-A and Trensor-C circuits showed no increase in mode fluorescence (Figure 4A, Supporting Figures 1 and 2), as per our LOD definition. HeLa cells harboring Trensor-T showed an increase in mode fluorescence upon incubation with TSA, and no similar increase when incubated with HCS, TCS, or 4-HT (Figure 4A, and Supporting Figure 3).

Taken together, we suggest the responsiveness of the Trensor-T circuit treatment with polyplexed pDNA treatment (Figures 1(1) and 3(1)), and to treatment with small molecules alone (Figure 4B), validates the Trensor concept for rapidly evaluating candidate transfection methods and materials. This work also suggests two major avenues for future research and development. First, dissecting the signal transduction pathway whereby the input signal leads to stimulation of the transgenic TRA2B promoter (Figure 4B), and second, trialing a larger panel of promoters to identify improvements in “Trensor” performance. Experimental steps that could assist both these research avenues include obtaining clonally derived populations from the polyclonal parental Trensor-T population, trialing a greater range of transfection agents, plasmids, and transfection-influencing small molecules, and also applying all of these approaches in different mammalian cell lines.

The Trensor-T circuit reported here represents a potentially powerful tool for real-time monitoring of cellular responses during transfection, potentially revolutionizing the process of optimizing transfection conditions in both research and therapeutic settings. Insights from using Trensor-T in this way could potentially help researchers understand and improve gene therapy and particle-based mRNA delivery. Such insights could pave the way for the development of more targeted and efficient treatments and vaccines.

## Data Availability

Raw data available on request.

## Supporting Information Available

Histogram plots of the raw flow cytometry data analyzed in Figure 4 (PDF)
