# Supplementary material for: Genetically Encoded Trensor Circuits Report HeLa Cell Treatment with Polyplexed Plasmid DNA and Small-Molecule Transfection Modulators
Source: ACS Synth Biol. 2024 Sep 6;13(10):3163–72. doi: 10.1021/acssynbio.4c00148 (PMC11494703; doi:10.1021/acssynbio.4c00148)
Supplement: Supplementary file 1 — sb4c00148_si_001.pdf [file sb4c00148_si_001.pdf]

Supporting Information

Genetically-encoded Trensor circuits report HeLa cell treatment with polyplexed plasmid DNA and small-molecule transfection modulators

Chileab Redwood-Sawyerr, Geoffrey Howe, Andalucia Evans Theodore, Darren N. Nesbeth*

Department of Biochemical Engineering, University College London, Bernard Katz Building, London WC1E 6BT, UK.

*Corresponding author: Department of Biochemical Engineering, University College London, Bernard Katz Building, London WC1E 7JE**.** Tel: +44 (0)20 7679 9582, Fax: +44 (0) 207 916 3943, Email: d.nesbeth@ucl.ac.uk

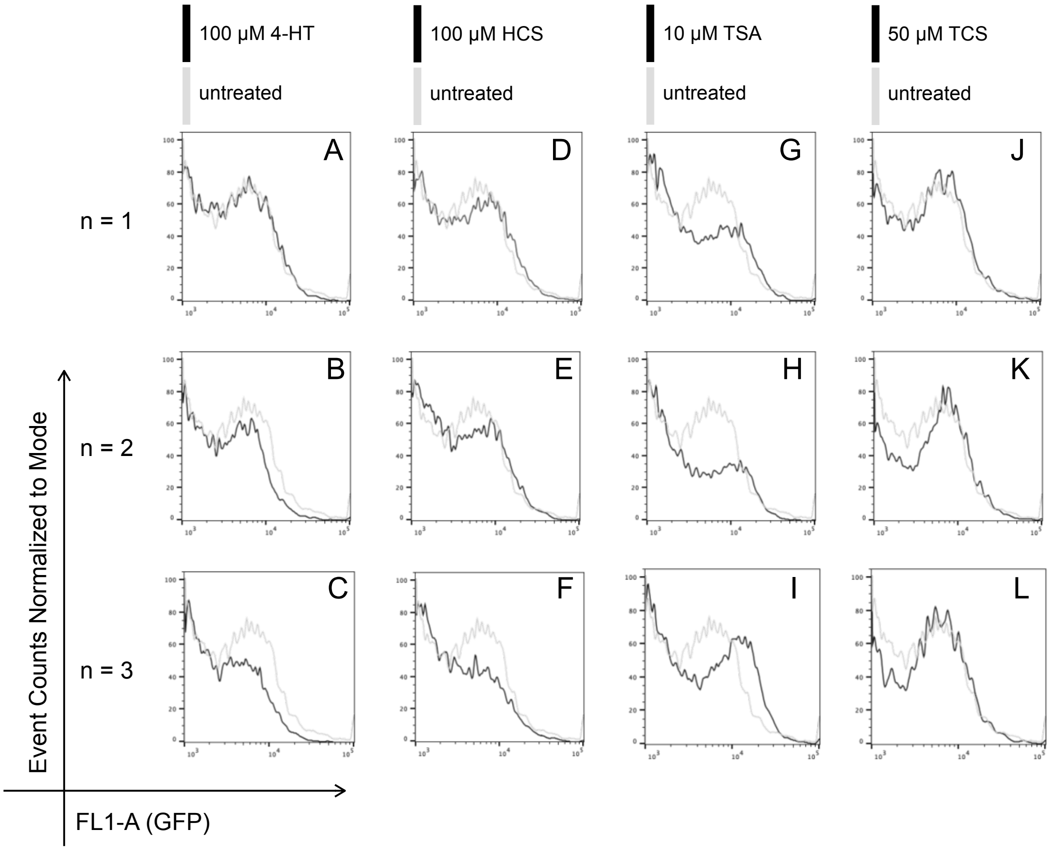

Figure S1. HeLa cells harbouring Trensor-A circuit unresponsive to 4-HT, HCS, TSA, and TCS. The HeLa Trensor-A cell line was separately incubated with four different chemicals as described in Materials and Methods. Fluorescent profiles from three independent repeats of the procedure (indicated by n=1, 2 and 3 on the left of the figure) were plotted in columns. The level of fluorescence in untreated cells is show in the grey fluorescence profile in each data plot, and the level in treated cells in the black fluorescence profile. Y and X axes labels for all plots are indicated in the bottom right of the figure. Incubation with 4-HT, HCS, TSA, and TCS, column are plotted, with supplement concentrations indicated at the top of each column, along with bars as a key for the grey and black data profiles in each column.

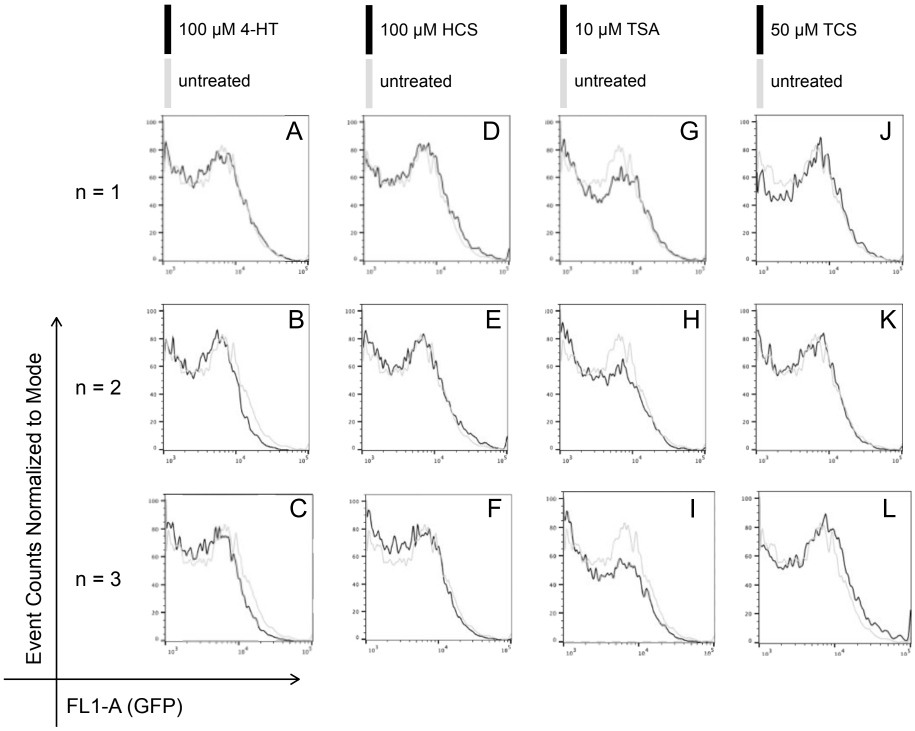

Figure S2. HeLa cells harbouring Trensor-C circuit unresponsive to 4-HT, HCS, TSA, and TCS. As described in Materials and Methods, the HeLa Trensor-C cell line was separately incubated with the same four chemicals as in Supplementary Figure 1. Resulting data were plotted in the same manner as described in Supplementary Figure 1.

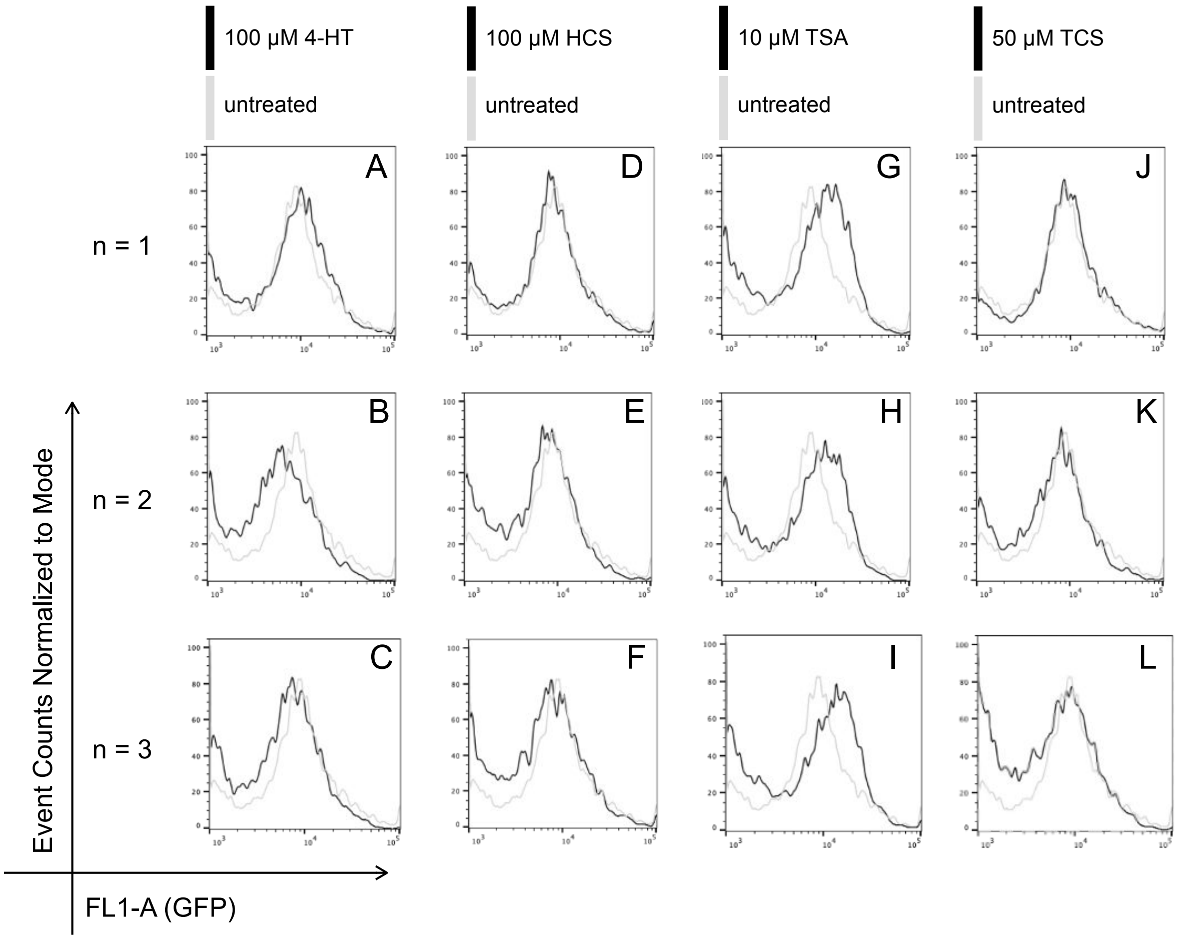

Figure S3. HeLa cells harbouring Trensor-T circuit respond to TSA but not 4-HT, HCS or TCS.. As described in Materials and Methods, the HeLa Trensor-T cell line was separately incubated with the same four chemicals as in Supplementary Figure 1. Resulting data were plotted in the same manner as described in Supplementary Figure 1. Notably, TSA treatment (G-I) caused an increase in modal fluourscence compared to untreated cells, in all of n=3 independent repeats of the procedure.
